# Investigating the association of self‐image and brain maps in medical students: A quantitative electroencephalography study

**DOI:** 10.1002/pcn5.70105

**Published:** 2025-05-06

**Authors:** Hamid Dehghan, Arvin Hedayati, Arash Mani

**Affiliations:** ^1^ College of Medicine Shiraz University of Medical Sciences Shiraz Fars Iran; ^2^ Research Center for Psychiatry and Behavior Science, Hafez Hospital Shiraz University of Medical Sciences Shiraz Fars Iran; ^3^ Substance Abuse and Mental Health Research Center, Hafez Hospital Shiraz University of Medical Sciences Shiraz Fars Iran

**Keywords:** brain map, gender, medical students, prefrontal lobe, QEEG, self‐image

## Abstract

**Aim:**

Self‐image, conceived as one's mental blueprint and a composite of thoughts, is among the hotly debated topics in psychology. Exploring the brain's structure, functionality, and physiology has also proven crucial in understanding one's self‐image. In this vein, the current study aimed to examine the relationship between quantitative electroencephalography (QEEG) findings and medical students' self‐image.

**Methods:**

To measure self‐image, the Offer Self‐Image Questionnaire and, for the QEEG findings, a Mitsar EEG‐202 device were used. To analyze the QEEG findings, the FDA‐approved Neuroguide software was utilized.

**Results:**

In the first phase, out of the 93 questionnaires returned, the maximum score, on a scale of 1 to 6, was 5.52 and the minimum was 2.36. Hence, it could be argued that the medical students who took part in this study had relatively high self‐images. Results, however, showed no significant difference between the two genders regarding either their overall self‐image score or any of its subcategories. In the second phase, the QEEG analyses of high‐ and low‐self‐image students suggested a statistically significant difference in their θ signal of the frontal lobes. Further analysis indicated that the difference was not localized to any single lead or lobe, but pertained to the overall function of the prefrontal cortices.

**Conclusion:**

The relationship observed between medical students' self‐image and their brain θ waves could contribute to a better understanding of people's cognitive functions.

## Graphical abstract

This study investigated the possible relationship between medical students' self‐image and their brain maps. To this end, brain maps of high‐ and low‐self‐image students were compared. Findings suggested a statistically significant difference in the θ signal of the two groups' frontal lobes. Further analysis inidcated that the difference was not localized to any single lead or lobe, but pertained to the overall function of the prefrontal cortices.

## INTRODUCTION

For many years now, self‐image and its role in a person's life have sparked research interest in psychology. Self‐image is the sum of thoughts, feelings, and beliefs which individuals hold in their mind about themselves. This internal image is a result of the person's personality, their environment, and mental processes. It enables its holder to recognize themselves as a separate individual from everyone else.^1^, ^2^, ^3^ It is generally believed that a high self‐image can boost one's chances of success.^4^ When self‐image is formed, it will act as a mental blueprint in the person's life, regulating their thoughts and actions, generally without questioning the underlying cause; however, self‐image is not constant, and is believed to shift and evolve through the person's life.^2^, ^5^, ^6^

**Figure 1:**
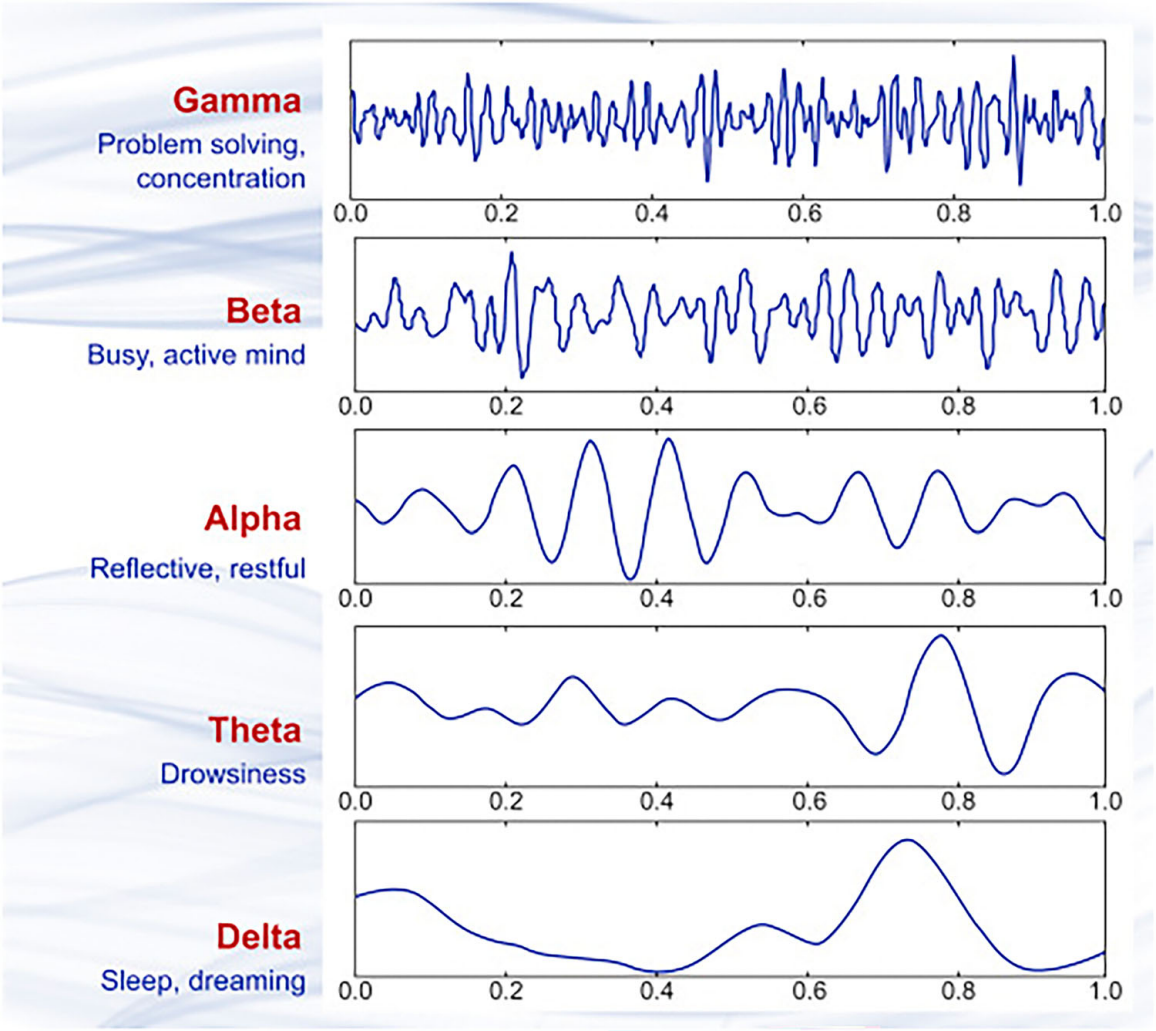
Quantitative electroencephalography (QEEG) signals as analyzed by software. QEEG is generally a complex recording of many waves. The measurements are fed through software that analyzes and pulls apart these different waves and categorizes them based on the wavelengths. The results shown are the δ (longest wavelength), θ, α, and β (shortest wavelength) waves, which are then used to examine different brain functions.

Given that the brain is the central organ to a person's feelings and behavior,^7^ one could convincingly argue that brain structure and chemistry might dictate any part of its owner's mentality, including their self‐image. In this regard, one of the instruments that has been used for the study of the brain is quantitative electroencephalography (QEEG), which records the brain's basal activity or, in some cases, its response to stimulants. This feature of QEEG deems it an eminently suitable tool in the investigation of the brain, and its function and physiology.^8^ The output of QEEG in a resting subject is generally composed of complex waves which are then analyzed by software and divided into sine wave spans with different wavelengths, namely, δ, θ, α, and β (Figure 1)^9^ in most cases.^10^ A brain map, on the other hand, is the result of a computer analysis of the QEEG findings, which displays a visual interpretation of the brain waves.

To the best of the present researchers' knowledge, to date, no previous study has explored the relationship between one's self‐image and brain function. In addition, most studies considering either of these variables have focused on patients and pathologies. To fill this lacuna, this study set out to investigate the possible differences (or lack thereof) in the QEEG findings of healthy medical students with high and low self‐images. While no aspect of one's personality can be attributed to a single region of the brain, given that past research suggested the prefrontal cortex as the most likely central brain region accounting for personality traits,^11^ the current study focused on this region. As a subsidiary goal, the study intended to compare female and male students' self‐image to uncover potential differences between the two genders. In line with these objectives, it set out to seek answers to the following research questions:

1. Is there any statistically significant difference between high‐ and low‐self‐image medical students in terms of the QEEG findings of their prefrontal cortices?
2. Is there any statistical difference between female and male medical students regarding their self‐image?

## BACKGROUND TO THE STUDY

This section is divided into two parts. First, studies on brain mapping are reported and then the background to self‐image research is reviewed.

### Studies on brain mapping

The potential of QEEG for investigating the differential diagnoses of a condition has been examined in a few studies. In one of the early studies, Jacobson et al. set out to test the efficacy of QEEG in differentiating between encephalopathy, delirium, and dementia. For this purpose, they chose 34 patients aged between 57 and 93 years and performed standard 17‐channel EEG on them. For each patient, the DSM‐5‐based diagnosis at the time of discharge was picked as the basis for comparison and a stepwise discriminant analysis was adopted to match specific brain map findings to the subject's condition. It was shown that mini‐mental state examination factors and relative force in α band frequencies were most useful in diagnosing encephalopathy. Furthermore, θ activity, relative force of δ, and the total score of the brain map were the most important in telling delirium apart from psychosis. The researchers emphasized that brain mapping has the potential to aid in the diagnosis of these conditions in the early stages.^12^

In a similar study in 1999, Monastra et al. investigated the potential of QEEG for diagnosing attention deficit hyperactivity disorder (ADHD). They recruited 482 participants whose age ranged from 6 to 30 years and assigned them, based on their diagnosis, to groups of combined ADHD, inattentive ADHD, and control. The brain maps of the ADHD participants, irrespective of the type, showed significant maturation effects in cortical arousal as well as signs of cortical slowing. These results suggested that brain mapping can be an important tool in ADHD diagnosis.^13^

More recently, in 2019, an effort was made to evaluate the potential of EEG in diagnosing borderline personality disorder. Despite their similarities, borderline personality, post‐traumatic stress disorder, and bipolar and depression disorders are recognized as different entities by DSM‐5, yet missed and wrong diagnoses are not uncommon. In this research, Pop‐Jordanova et al. tried to analyze the characteristics of these patients' brain maps. They found that the participants' brain function was statistically similar to that of healthy individuals, with the only difference being in the lower bands, δ and θ, which showed much lower frequencies and coherence. The researchers therefore emphasized that this field merits further research.^8^

In another study in 2021, Weon et al. compared brain maps of couples who suffered from domestic violence with those of healthy ones. For this purpose, they selected two couples in which one individual subjected the other to intimate partner violence, as well as a healthy couple as the control. The brain maps of all six individuals were obtained and compared. The results showed that the afflicted couples had stronger δ, θ, and β waves in their frontal lobes compared to the controls, which was probably a sign of emotional stress, anger, and failure to communicate. These researchers proposed that brain wave training through neurofeedback could be a promising intervention to help these couples to tackle their issues.^11^

### Studies on self‐image

A surging number of studies have examined self‐image and its role in a person's life. Some of these studies focused on the impact of self‐image on success whereas some others brought attention to its effects on diseases and their manifestations. As an example, in one of the early studies in 1993, the relationship between self‐image and eating disorders was the subject of inquiry in Germany. In this study, Steinhausen and Vollrath surveyed 60 adolescents who were followed for long‐term sequalae of these conditions and evaluated their self‐image using the Offer Self‐Image Questionnaire (OSIQ). Then, they compared these results to the mean scores of German adolescents using *t*‐test. The researchers found that the participants' scores deviated from those of normal adolescents; however, in those who underwent psychotherapy, both self‐image and general health improved.^14^

In 2003, Erkolahti et al. attempted to examine the relationship between self‐image and depression in adolescents. They recruited 1054 8th‐grade students and evaluated their self‐images using the OSIQ. As for the symptoms of depression, the participants were given the children's depression inventory test, and the results were compared for each individual using the Pearson correlation test. The researchers observed that there was a strong relationship between self‐image and the number and severity of depression symptoms in those participants. Moreover, the girls had lower self‐image levels and a stronger correlation between self‐image and depression.^15^

Two years later, in 2005, Erkolahti and Ilonen investigated the self‐image of students with type I diabetes mellitus or rheumatoid arthritis, and its impact on their success in education. For this purpose, they selected 69 students and gave them the OSIQ questionnaire. The results were then cross‐examined with their grades. The researchers found that there was no significant difference between students with mild symptoms and the healthy ones. However, compared to the control group, the correlation between self‐image and grades was less predictable in the tested group.^16^

Hintikka et al.^17^ tried to improve the self‐image of 13‐ to 18‐year‐old adolescents who had multiple attempts for suicide. The researchers chose 88 participants and after assessing their self‐image using the OSIQ questionnaire and a few other psychology tests referred them to the hospital of the University of Finland. After a 5‐month period of interventions, consisting of psychotherapy, exercise and group sessions, as well as medications, they were discharged and given the same tests and questionnaires again. The results showed that the self‐image of these teenagers had dramatically improved and even approached that of the normal population. Moreover, the participants felt less demotivated, reported having suicidal ideas significantly less often, and improved in different social activities.^17^

In 2013, Anderson et al. suggested the idea that a weak self‐image may be related to the probability of deliberate self‐harm in adolescents. Studies before that had demonstrated that these behaviors were related to adult individuals' social conditions. They chose a sample of 113 high‐school students and asked them to fill out a 16‐item questionnaire from the Deliberate Self‐Harm Inventory. For their self‐image, they were given the structural analysis of social behavior (SASB) questionnaire. The SASB questionnaire is a model devised in 1974 by Benjamin, based on the Interpersonal Theory, which states that a person's self‐image is formed through their relationships with others. The researchers performed a Spearman's correlation test on the data which showed that deliberate self‐harm was in fact more common among those with weaker self‐images. Moreover, these participants scored higher in terms of self‐suppression and self‐ignorance and rejection, and lower in terms of self‐acceptance and self‐expression.^18^

In a more recent study, Di Blasi et al.^3^ investigated the relationship between self‐image and social anxiety disorder. Patients with social anxiety disorder avoid groups and find it hard to communicate with others. Overall, 1305 high‐school students participated in the study and completed the OSIQ and social interaction anxiety scale questionnaires for self‐image and level of social anxiety, respectively. After the results were interpreted, it was found that social anxiety was directly correlated with a person's level of self‐image.^3^

In summary, to the best of the present researchers' knowledge and as reviewed in previous sections, a host of empirical studies have so far been conducted on either brain mapping or self‐image, but no single study has been devoted to investigating the possible relationship between these two important variables. In an attempt to bridge this gap, this study endeavored to shed light on this under‐researched area of inquiry to serve as a stepping stone for more studies in this area.

## METHOD

### Sampling procedures and the participants

Prior to embarking on this study, ethical approval was sought from the university's Institutional Review Board. After being reviewed by all committee members, permission was granted to commence the study under the ethical approval code of IR.SUMS.MED.REC.1401.355. Then, sampling was done in two phases. First, a convenience sampling procedure was employed through which 93 medical students (female = 42, male = 51) who were available to the researchers were asked to fill out the self‐image questionnaire. These students were enrolled in the basic sciences course at a public university of medical sciences in one of the large cities of Iran. Their ages ranged from 18 to 25 years, with an average of 19.88. They were asked to fill out the questionnaire as sincerely and honestly as possible and include their contact information should they meet the criteria for the second phase of the study. The students' self‐image scores were then computed, both generally and in each subcategory defined in the questionnaire using SPSS software.

In the second sampling phase, the participants whose scores were one standard deviation above as well as the ones whose scores were one standard deviation below the mean were recruited as high‐ (*N* = 14, female = 4, male = 10) and low‐self‐image (*N* = 13, female = 7, male = 6) students, respectively, and the remaining students were excluded from this phase of the study. Next, the researchers contacted the recruited participants and invited them to perform a QEEG in the local psychiatric hospital, where the second researcher served as the department head. To compensate for their cooperation, they were offered a minor monetary incentive. It was imperative to exclude the participants who had known psychiatric disorders at this stage, but none of them reported such problems, and hence all 27 students identified were considered as the participants of the second phase.

In this phase, after obtaining the participants' informed consent for the process, a QEEG was recorded with the help of hospital technicians who were sufficiently experienced in the task. The recordings were done in a quiet room and for a period of at least 10 min. The participants were asked to remain calm and motionless, and stare at a single point during the process. The recordings were then fed into the Neuroguide software and the artifact rejection process was carried out by the technicians. The tolerated reliability of the artifact rejection, provided by the software, was set at 90% for better and more reliable results.

### Instruments

To measure the students' self‐image, the OSIQ questionnaire, first developed by Offer and his colleagues in 1961, was utilized. This questionnaire consists of a total of 99 questions and assesses six different aspects of self‐image as follows: social self, sexual self, coping self, psychological self, familial self, and individual values. The questionnaire has been examined regarding the overall score, inter‐ and intraclass correlations, and its reliability and validity have been established by its creators.^19^ Its items are based on a Likert scale of 6, according to which, an answer of 1 would mean “describes me very well” while 6 would mean “does not describe me at all”. In this study, the Persian translation of this questionnaire by Alimorad and Yazdani^20^ was used to avoid the target language acting as a barrier to students' understanding the questions well. Before translating it, they excluded seven items perceived to be culturally inappropriate for the context of Iran from the original questionnaire. To ensure the reliability of the translation, they examined it against the original one through back‐translation and checked its validity and reliability.^20^

According to Offer et al.'s guide for the questionnaire, selecting 1, 2, or 3 for any given item would mean that the participant agrees with that statement while choosing 4, 5, or 6 would mean the other way round. The point that must be borne in mind is that some items necessitated a reverse scoring procedure, which was applied following the guidelines. Having calculated the overall scores, the researchers divided the scores by the number of items so that the final scores would fall on a scale of 1–6, in which higher scores were indicative of higher self‐image and vice versa. Next, the data were fed into SPSS 26 software and the means and standard deviations of female and male students' scores were calculated, for self‐image on the whole and also in each subcategory. Finally, an independent samples *t*‐test and a multivariate analysis of variance (MANOVA) test were run to compare the two genders in terms of their overall self‐image scores and in each subcategory, respectively.

### The QEEG

**Figure 2:**
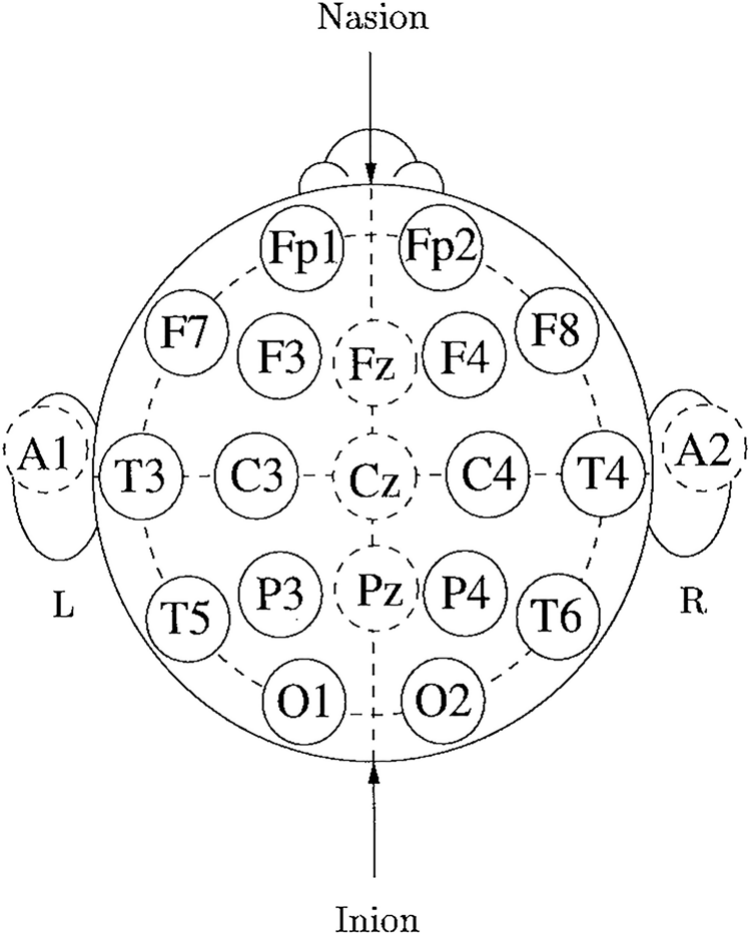
Quantitative electroencephalography records signals using special devices that have a certain number of electrodes. These electrodes are placed on different locations on the subjects' scalps. A sample diagram of electrode placement, which was used for the purposes of the current study, is shown.

For the QEEG recording, a Mitsar EEG‐202 device was used along with a 19‐electrode cap, which was in line with the international standard system of 10–20 electrodes (Figure 2),^21^ and two‐ear‐attached electrodes were used for reference. Before the recording day, the subjects were instructed about the preparations and asked to get a good night's sleep to minimize any possible irregularities. Recording was done at a frequency of 256 Hz and with open eyes and a steady gaze. The decision to record the EEGs with open eyes was based on the assumption that this approach would reduce α wave activity, which is generally concerned with arousal states.^22^ Additionally, recordings were conducted around the same time of day to reduce variability. Finally, each recording was analyzed using the FDA‐approved Neuroguide software. In this study, the 2.9.0.0 version of the software was used, which was the latest at the time.

**Figure 3:**
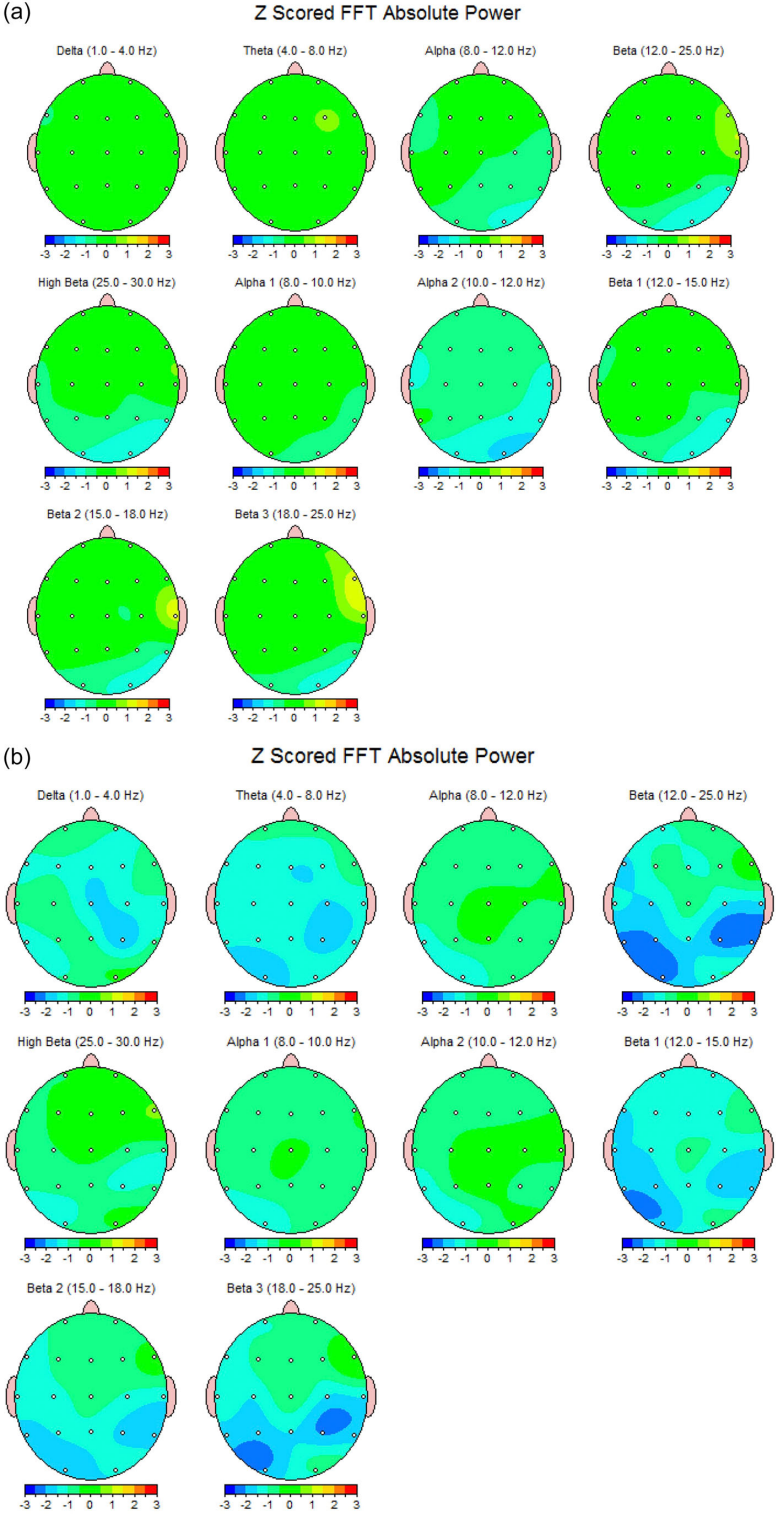
The quantitative electroencephalography software also provides visual representations of the brain waves. Different wave amplitudes are shown with different colors for easier understanding. The final illustrations are called brain maps and are used for interpretation. Two sample brain maps from the current study are portrayed in this figure for comparison; one map belongs to a subject with a high self‐image level and the other to a subject with a low self‐image level. (a) belongs to a high self‐image subject and (b) to a low self image one.

The current researchers decided that the best paramter for the purposes of the study was the absolute power of the waves, which was recorded in microvolts. Neuroguide compares all the data it is fed about every participant, with the mean of their exact age group and therefore presents a standardized *z* score for each. Moreover, as mentioned earlier, the region of the brain that is in charge of the “self” is the prefrontal cortex,^11^ whose activity is picked up by the six leads of the device lying directly above it. These leads are Fp1, F3, and F7 for the left lobe, and Fp2, F4, and F8 for the right (Figure 2).^21^ It was therefore decided to concentrate on the output of these six leads. A MANOVA test was run for each of the recorded wavelengths and the outputs were closely examined. Two brain map samples belonging to a high‐ and a low‐self‐image participant are displayed in Figure 3.

## RESULTS

In the sections that follow, the results of the data analysis are reported in an attempt to answer the research questions posed at the beginning of this study.

### Descriptive statistics regarding the participants' self‐image

As shown in Table 1, the maximum and minimum self‐image scores were 5.52 and 2.36 (out of 6), respectively. In this regard, the mean was 4.30 and the standard deviation was calculated to be around 0.54. Based on this finding, it could safely be inferred that the medical students who took part in this study enjoyed relatively high self‐image scores, which were almost evenly distributed (SD = 0.54).

**Table 1:** Descriptive statistics of the participants' overall self‐image.

	Minimum	Maximum	Mean	Standard deviation
Score	2.36	5.52	4.30	0.54

Table 2 displays the descriptive statistics of each of the self‐image subcategories. As observed in the table, the highest mean belonged to individual values (4.75) whereas the lowest was obtained for the sexual self (3.75). Nevertheless, apart from the sexual self, the obtained mean for all other subcategories exceeded 4, which, as mentioned above, could imply that overall, these participants' self‐image was found to be relatively high. Considering minimum scores, it could be seen that psychological and familial selves displayed minimum scores <2 while the rest exhibited minimum scores more than 2. Despite not being one of the objectives of this study, this finding could be a fruitful area of enquiry in future studies.

**Table 2:** Descriptive statistics of different subcategories of the self‐image.

	Minimum	Maximum	Mean	Standard deviation
Psychological self	1.91	5.41	4.03	0.68
Social self	2.75	5.62	4.21	0.60
Sexual self	2.20	5.4	3.75	0.74
Familial self	1.76	5.76	4.52	0.80
Coping self	2.41	5.79	4.49	0.66
Individual values	2.66	6.00	4.75	0.71

Finally, the relatively small standard deviations obtained in this study suggest that the participating students' self‐image, even in terms of its subcategories, was almost evenly distributed.

### Comparing female and male students' self‐image

To compare the self‐image scores of the two genders, an independent samples *t*‐test was run. Prior to conducting the test, the assumption of normality was assessed using Kolmogorov–Smirnov and Shapiro–Wilk tests. Specifically, for male students, the Kolmogorov–Smirnov and Shapiro–Wilk tests (P = 0.200 and 0.822, respectively) indicated no violation of normality. For female students, the Kolmogorov–Smirnov test (P = 0.037) also suggested a normal distribution, whereas the Shapiro–Wilk test (P = 0.037) indicated a slight departure from normality. Nevertheless, given the moderate sample sizes (*N* = 42, 51), it can be reasonably concluded that the assumption of normality was sufficiently met for conducting the independent samples *t*‐test. The results of the *t*‐test showed a mean score of 4.24 (SD = 0.64) for female and 4.35 (SD = 0.45) for male students while no statistically significant difference was observed between the two genders (*t* (91) = −0.982, P = 0.329, two‐tailed; Tables 3 and 4).

**Table 3:** Descriptive statistics of self‐image for the two genders.

Gender	*N*	Mean	Standard deviation	Standard error of the mean
Female	42	4.24	0.64	9.16732
Male	51	4.35	0.45	5.84680

**Table 4:** Independent samples *t*‐test results.

		Levene's test for equality of variances		*t*‐test for equality of means						
		*F*	Significance value	*t*	*df*	Significance value (2‐tailed)	Mean difference	Standard error difference	95% confidence interval of the difference	
									Lower	Upper
Score	Equal variances assumed	4.353	0.040	−0.982	91	0.329	−10.32493	10.51842	−31.21849	10.56863

To obtain a more complete picture of the participants' self‐image, a one‐way between‐groups MANOVA test was run to compare male and female students' self‐image in terms of its different subcategories. Prior to conducting the test, preliminary assumption testing was performed for checking normality, linearity, univariate and multivariate outliers, homogeneity of variance–covariance matrices, and multicollinearity and no serious violations were noticed (Mahalanobis distance value = 16.47 < 22.46, Box's test=0.935 > 0.001, Levene's test for all variables>0.05). As can be seen from Table 5, the MANOVA results showed no statistically significant difference between males and females on the combined dependent variables (*F*(6, 86) = 2.034, P = 0.07, Wilks' *λ* = 0.876, partial *η* ^2^ = 0.124). This finding indicates that female and male students who took part in this study did not differ in terms of self‐image subcategories.

**Table 5:** Multivariate analysis of variance results.

	Value	*F*	Hypothesis *df*	Error *df*	Significance value	Partial *η* ^2^
Wilks' *λ*	0.876	2.034	6.000	86.000	0.07	0.124

### Comparing brain maps of high‐ and low‐self‐image students

As the main motive behind conducting this study, the brain maps of prefrontal cortices of high‐ and low‐self‐image students were compared using the MANOVA test. Preliminary assumptions were checked to ensure that all of them were met. Table 6 displays the results of these tests.

**Table 6:** Multivariate analysis of variance results for the participants' brain maps.

Wavelength	Wilks' *λ* value	*F*	Hypothesis *df*	Error *df*	Significance value	Partial *η* ^2^
δ	0.60	2.20	6.00	20.00	0.085	0.398
θ	0.53	2.95	6.00	20.00	0.031	0.470
α	0.70	1.37	6.00	20.00	0.272	0.292
β	0.912	0.32	6.00	20.00	0.919	0.088
High β	0.94	0.19	6.00	20.00	0.973	0.056

As could conspicuously be observed in the above table, the θ wavelength is the only variable which exhibited a statistically significant difference between low‐ and high‐self‐image students (*F*(6, 20) = 2.95, P = 0.031, Wilks' *λ* = 0.53, partial *η* ^2^ = 0.470) and the rest of the variables did not show any significant difference between the two groups (δ: *F*(6, 20) = 2.20, P = 0.085, Wilks' *λ* = 0.60, partial *η* ^2^ = 0.398; α: *F*(6, 20) = 1.37, P = 0.272, Wilks' *λ* = 0.70, partial *η* ^2^ = 0.292; β: *F*(6, 20) = 0.32, P = 0.919, Wilks' *λ* = 0.912, partial *η* ^2^ = 0.088; high β: *F*(6, 20) = 0.19, P = 9.973, Wilks' *λ* = 0.94, partial *η* ^2^ = 0.056).

This finding led the researchers to perform some follow‐up analyses on the study leads in the θ band. On the whole, the mean of the *z*‐scores for leads Fp1, Fp2, and F8 were higher in the low‐self‐image group whereas those for leads F3, F4, and F7 were higher in the high‐self‐image group (Table 7). This shows that on average, θ signals were slightly stronger in the low‐self‐image group in the former leads, and vice versa in the latter ones. However, as is clear from Table 8, none of these findings in the six examined leads were statistically significant (Significance value = 0.904, 0.247, 0.257, 0.591, 0.368, 0.713 > 0.05). This result shows that the difference was not in the function of any single spot of the brain, but rather in the whole prefrontal cortices, as a single functioning system.

**Table 7:** Descriptive statistics of different leads in the θ band.

Dependent variable	Self‐image level	Mean	Standard error	95% confidence interval	
				Lower bound	Upper bound
FP1LE θ	Low	−0.172	0.209	−0.603	0.258
	High	−0.208	0.201	−0.623	0.207
F3LE θ	Low	−0.399	0.216	−0.844	0.045
	High	−0.044	0.208	−0.473	0.384
F7LE θ	Low	−0.381	0.221	−0.836	0.075
	High	−0.024	0.213	−0.463	0.415
FP2LE θ	Low	−0.088	0.181	−0.460	0.285
	High	−0.224	0.174	−0.583	0.134
F4LE θ	Low	−0.390	0.248	−0.900	0.120
	High	−0.075	0.239	−0.566	0.416
F8LE θ	Low	0.064	0.247	−0.444	0.572
	High	−0.064	0.238	−0.553	0.426

**Table 8:** Comparison of the θ band in each lead.

Source	Dependent variable	Type III sum of squares	*df*	Mean square	*F*	Significance value	Partial *η* ^2^
Self‐image level	FP1LE θ	0.009	1	0.009	0.015	0.904	0.001
	F3LE θ	0.849	1	0.849	1.403	0.247	0.053
	F7LE θ	0.857	1	0.857	1.347	0.257	0.051
	FP2LE θ	0.126	1	0.126	0.296	0.591	0.012
	F4LE θ	0.669	1	0.669	0.840	0.368	0.032
	F8LE θ	0.109	1	0.109	0.138	0.713	0.005

## DISCUSSION

Contrary to previous research 3, 15, 16, 17, 18, 23, the results of the *t*‐test and MANOVA indicated that the participating students enjoyed relatively high self‐images in terms of both their overall scores and the scores pertaining to different subcategories. Nevertheless, this discrepancy could be justified on the grounds that the participants of this study were healthy medical students while the aforementioned studies focused on patients suffering from pathological conditions (e.g., deliberate self‐harm in adolescence, social anxiety disorder, depression, suicide attempts, type I diabetes mellitus or rheumatoid arthritis, and eating disorders among others). Although the current researchers did not have any prior assumption regarding the possible self‐image level of the study sample, this finding may not be unexpected given that in the context of Iran, senior high‐school students have to win a tough competition to be accepted as students of medicine, especially at public universities, therefore those who are accepted are usually the most gifted, motivated, and hard‐working students, who most likely possess high self‐images. However, this is unlikely to present a significant challenge to the second phase of the study because the students recruited for this phase demonstrated distinctly high and low self‐image levels, as determined by the means and standard deviations of their responses to the questionnaire items. Furthermore, no statistically significant difference was observed between male and female students in terms of either their overall self‐image scores or its subcategories. This finding contradicts results of some previous studies (e.g., Erkohlati et al., 2003) which found females to have lower self‐images compared to their male counterparts. However, as mentioned above, this contradiction might be due to the nature of samples examined in those studies (i.e., depressed adults) in comparison to the sample recruited in this context, who were healthy young students of medical sciences.

Finally, a statistically significant difference was found in the brain maps of the participants with high and low self‐images. More specifically, these two groups were different in the θ span of the QEEG of their frontal lobes. This finding lends support to those of some other similar studies. For example, in 2019, Pop‐Jordanova et al. studied patients with borderline personality disorder using QEEGs. They found the only difference with healthy individuals was in the lower bands, δ and θ, which showed much lower frequencies and coherence. Additionally, in a more recent study in 2021, Weon et al. compared the brain maps of couples who suffered from domestic violence to those of couples who did not have the same problem, which indicated stronger δ, θ and β waves in the frontal lobes of the afflicted couples in comparison to the controls. These findings might be suggestive of the likely role played by slower brain waves in determining an individual's beliefs and even their personality. Despite being promising, given the limitations of different studies, such findings merit further examination and investigation by future researchers and thereby in the current state no definitive conclusion could be drawn. Nevertheless, this study and other similar ones could pave the way for more studies on the possible role of brain waves in determining people's personality and characteristics.

## CONCLUSION

This study uncovered a relationship between medical students' self‐image and their brain θ waves. Hence, high‐ and low‐self‐image medical students exhibited a statistically significant difference in their θ waves. However, this finding must be approached with some caution because we do not yet have a clear idea of the inner workings of the brain. Nevertheless, the results of such studies could help us form a better understanding of people's cognitive functions. In this regard, we can entertain the idea of exploiting advances in novel therapeutic methods such as reverse neurofeedback to improve current interventions or create new possibilities in the treatment of psychological disorders in general and low self‐image in particular. This effect is not at all limited to pathologies, however, and one could imagine how altering and improving one's mental state, by boosting self‐image, could help to improve quality of life. On the other hand, given the importance of self‐image and its impact on educational success,^20^ responsible authorities need to consider it when passing laws and making decisions for school and university systems in general, and universities of medical sciences in particular.

Despite its seemingly promising findings, this study, not unlike other studies, was not without some unavoidable limitations. The most serious limitation is the convenience sampling procedure employed, which could question the generalizability of its findings. Furthermore, the sample was homogeneous, consisting of medical students, and its size was rather small due to practical issues relating to QEEG recording. Given that quantitative studies that opt for generalizability of findings strive to draw large representative samples, it is suggested that future researchers attempt to replicate this study using larger and more representative samples to either confirm or refute current findings. Another point worth mentioning is that this study only focused on the frontal lobe and QEEG recordings with open eyes. It is highly recommended that further studies perform and compare the recordings with both open and closed eyes, and ideally consider the entire cortex if feasible.

Another important point that needs to be borne in mind is that in this study the gender of the participants was not considered while analyzing the data. It is highly likely that besides their level of self‐image, their gender also influences their brain waves. Future researchers need to either control the participants' gender as an extraneous variable or build it into the design of their studies. In the latter case, using more sophisticated statistical procedures, they will be able to examine the interactions between the participants' self‐image and their gender with respect to their brain waves in addition to the main effects of each of them separately.

Most importantly, given that the function of the brain is still poorly understood, utmost caution needs to be exercised while interpreting the results of this study. The brain is a complex organ consisting of multiple lobes and white and grey matter as well as different types of cells, different types of cells, and QEEG only opens a small aperture on them. Additionally, each person's social and psychological conditions may well affect their brain function, which is extremely difficult to control in studies. In fact, QEEG is only a functional recording of the electric function of neurons and further research is needed to shed light on the inner workings of the brain. Moreover, the relationship found between levels of self‐image and brain waves should not be considered a cause‐and‐effect relationship. Future researchers could conduct experimental or quasi‐experimental studies in an attempt to elucidate any possible causal relationships between the function of neurons and human characteristics. Finally, this study mainly focused on the brain area known to have the closest relationship to personality, which, despite limiting the scope of the study, could lend impetus to future research and more empirical studies on this rather neglected area of investigation.

## AUTHOR CONTRIBUTION

H. Dehghan, A. Hedayati, and A. Mani equally contributed to the completion of this study from its design and procedures, to data collection and analysis, preparing the report, and revising and finalizing the manuscript. Hence, all authors are equally accountable for all aspects of this study.

## CONFLICTS OF INTEREST STATEMENT

The authors declare that they have no competing interests. Although this study was partially supported by Shiraz University of Medical Sciences, study design and procedures were designed by the authors themselves. The mentioned support was received as a student grant and therefore the authors retain full publishing rights for the paper.

## ETHICS APPROVAL STATEMENT

Permission was granted to commence the study under the ethical approval code of IR.SUMS.MED.REC.1401.355 by the Committee of Ethics in Research in Shiraz University of Medical Sciences.

## PATIENT CONSENT STATEMENT

Informed consent was obtained from each participant before performing QEEGs. The authors have the rights to use and publish the data as long as the identity of each particular subject remains anonymous.

## CLINICAL TRIAL REGISTRATION

N/A

## Data Availability

The datasets generated and analyzed during the current study are available from the corresponding author on reasonable request.
